# Engineered extracellular vesicles: pharmacological barriers, engineering strategies, and translational opportunities

**DOI:** 10.20517/evcna.2026.85

**Published:** 2026-08-31

**Authors:** Jiao Xu, Zhi Li, Da Liu, Libo Zhao, Yongqing Li

**Affiliations:** ^1^The Center for Heart Development, College of Life Science, Hunan Normal University, Changsha 410081, Hunan, China.; ^2^Echo Biotech Co., Ltd, Beijing 102609, China.; ^#^These authors contributed equally to this work.

**Keywords:** Extracellular vesicles, engineered extracellular vesicles, drug delivery, therapeutic translation, bioengineering

## Abstract

Extracellular vesicle (EV) therapeutics are progressing from broad proof-of-concept studies toward product-specific engineering and clinically defined applications. Native EVs offer biological membrane functions and the capacity to transport diverse molecular cargoes, but their therapeutic performance is limited by low cargo stoichiometry, rapid mononuclear phagocyte system clearance, clearance-dominated biodistribution, and inefficient functional cargo release. This Review examines how EV engineering strategies are used to address these barriers. We compare approaches for cargo loading, circulation control, tissue- and cell-selective targeting, and intracellular delivery, while considering the immunogenicity and manufacturing risks introduced by engineering. We also assess recent preclinical and clinical progress, including programs that were paused or discontinued, and discuss the practical constraints of cost, supply chain, and cold-chain distribution. Current evidence suggests that engineered EVs are unlikely to replace established lipid nanoparticles or viral vectors across all applications. Their near-term value is more likely to arise in localized delivery, immune-microenvironment modulation, complex cargo delivery, and defined cell-targeting settings in which biological membrane functions provide a measurable advantage.

## INTRODUCTION

Extracellular vesicles (EVs) are lipid bilayer-enclosed nanoparticles actively secreted into the extracellular milieu and distributed across diverse biological fluids^[1]^. Once regarded primarily as metabolic byproducts, EVs are now recognized as mediators of horizontal molecular transfer and intercellular communication^[2-5]^. Early studies established heterogeneous populations of secreted vesicles, and subsequent work described multiple EV subtypes - including exosomes, microvesicles and apoptotic bodies - distinguished by their biogenesis, morphology and molecular composition^[6-9]^. Because these subtypes overlap in size, composition, and biogenetic features, individual particles are often difficult to assign to a single subpopulation [Figure 1].

**Figure 1:**
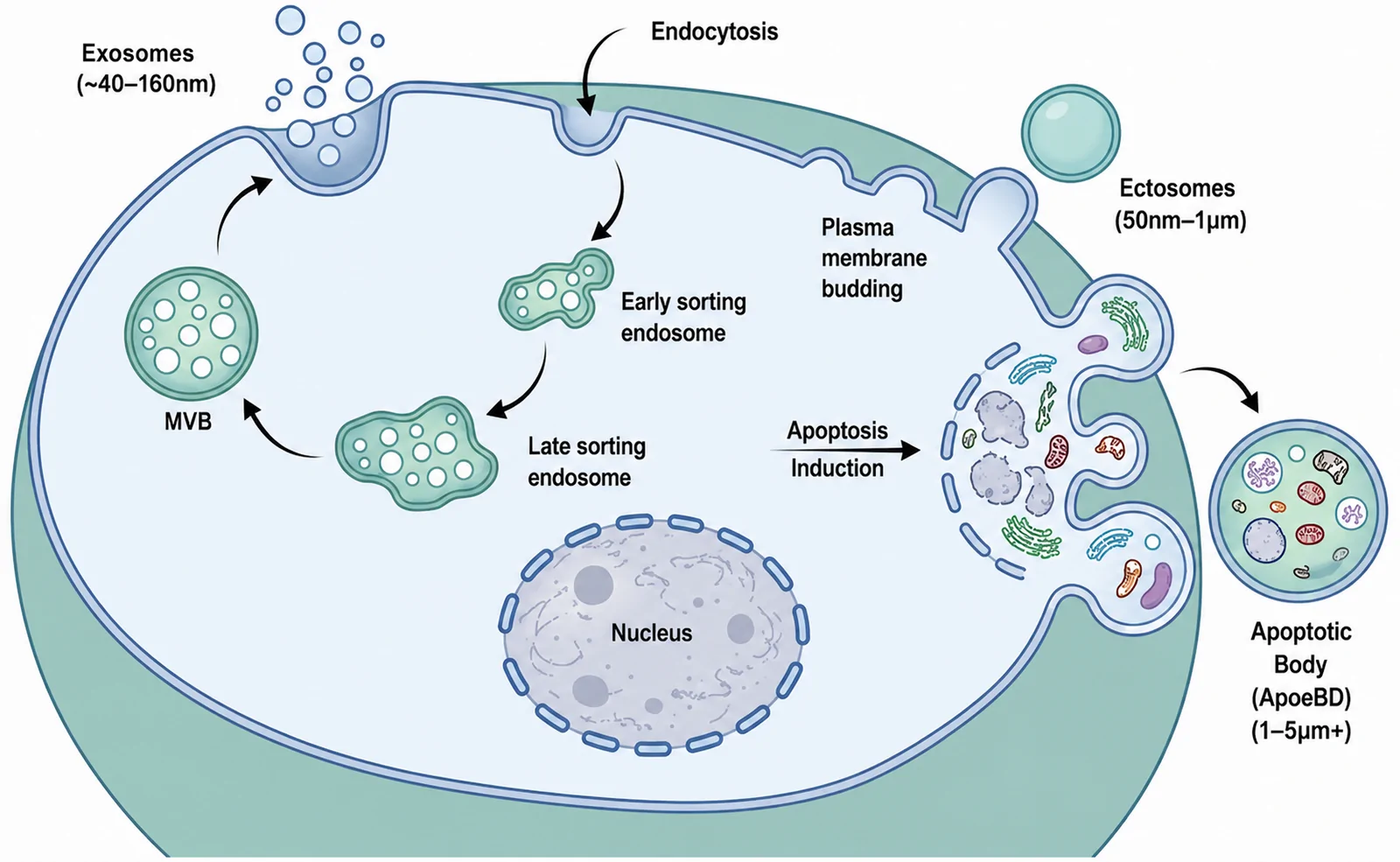
Biogenesis and classification of extracellular vesicle subtypes. Exosomes (approximately 40-160 nm) arise through the endosomal pathway, where early and late sorting endosomes mature into MVBs that fuse with the plasma membrane to release intraluminal vesicles^[6]^. Ectosomes or microvesicles (approximately 50 nm-1 μm) are generated by outward budding of the plasma membrane^[8,9]^. During apoptosis, membrane blebbing and cellular fragmentation generate ApoBDs (typically 1-5 μm or larger) that can enclose cytoplasmic components, organelles, and nuclear material. Substantial overlap in size and composition limits strict assignment of individua l extracellular particles to a single biogenetic pathway^[10,11]^. References^[8-11]^ support the scientific content depicted and were not used as sources for adaptation or reproduction. Created with BioRender. MVB: Multivesicular body; ApoBDs: apoptotic bodies.

The understanding of EVs evolved from cellular waste disposal to intercellular communication and therapeutic relevance. Vesicles released during reticulocyte maturation were first described in the late 1980s and were initially interpreted as part of a cellular clearance mechanism^[12,13]^. Subsequent studies showed that EVs transfer proteins, nucleic acids, lipids, and metabolites between cells^[1,14-16]^ and participate in antitumour immunity^[17-19]^, cardiovascular homeostasis^[1,20,21]^, neurodegenerative diseases^[22-25]^ and immune regulation^[26]^. These findings established EVs as a therapeutic modality at the intersection of biologics, cell-free therapies, and non-viral drug-delivery systems.

The appeal of EVs reflects a combination of endogenous membrane composition, membrane-protected cargo transport and the ability to carry several classes of bioactive molecules^[27]^. These features support their development as both native therapeutic products and engineered delivery systems. At the same time, EV heterogeneity and the difficulty of distinguishing bona fide EV-associated components from co-isolated extracellular particles create substantial challenges for product definition, analytical characterization, and regulatory evaluation^[28,29]^.

These challenges are also evident in the classification of EV-based therapeutics. Origin-based frameworks, such as the four therapeutic scenarios proposed by the International Society for Extracellular Vesicles (ISEV) in 2015^[28]^, provide conceptual clarity but are increasingly strained by the advent of multifunctional engineered EV products. For instance, the engEx® pipeline developed by Codiak BioSciences - comprising exoIL-12^TM[30]^, exoSTING^TM[31]^, and exoASO^TM^-STAT6^[32]^ - derives entirely from engineered producer cells. Yet, it encompasses fundamentally distinct pharmacological mechanisms, ranging from membrane-tethered recombinant cytokines^[33]^ and encapsulated small-molecule agonists^[34]^ to antisense oligonucleotide-mediated gene silencing^[35]^. Consequently, relying primarily on source-cell characteristics for classification risks obscuring the true pharmacological identity of the active therapeutic component.

As engineered EV modalities diversify, classification based solely on producer-cell origin becomes increasingly difficult to apply. In a 2025 draft document, China’s Center for Drug Evaluation discussed advanced-therapy products using a product-oriented framework that may distinguish cell-derived products from engineered delivery systems^[36]^. This proposal is specific to the Chinese regulatory context and should not be directly extrapolated to other jurisdictions, where EV products may be assessed under biologics, advanced-therapy, or drug-delivery frameworks according to their composition and mechanism of action. Internationally harmonized categories for engineered EV therapeutics have not yet been established.

## NATIVE EVs AS THERAPEUTIC AGENTS

The development of native EVs as therapeutic candidates emerged from changes in the understanding of how cell-based therapies exert their biological effects. Early models in regenerative medicine emphasized durable engraftment and direct tissue replacement by transplanted stem or progenitor cells^[37,38]^. However, subsequent studies reported therapeutic benefits despite limited cell persistence and minimal structural integration^[39]^. These observations shifted attention toward paracrine mechanisms, including the contributions of soluble factors and EVs. Further studies supported EVs as important, although context-dependent, mediators of the cell-free therapeutic activities associated with stem and progenitor cells^[40,41]^.

### From cell engraftment to paracrine mechanisms

These findings supported the paracrine hypothesis, which attributes much of the regenerative activity of stem cells to secreted bioactive factors rather than durable structural integration. Gnecchi and colleagues showed that conditioned medium from Akt-overexpressing mesenchymal stromal cells (MSCs) could reproduce the cardioprotective effects of the cells in myocardial injury models^[39]^. This result redirected attention from direct cell replacement toward soluble and vesicular components of the extracellular milieu.

Subsequent research across diverse disease models supported this view. Stem cell-derived secretomes were shown to promote angiogenesis, attenuate inflammatory responses, and stimulate endogenous tissue repair in cardiovascular, renal, and neurological settings^[41-45]^. These findings broadened regenerative medicine beyond direct cell replacement and identified secreted effectors as important contributors to therapeutic activity.

### EVs as principal mediators of intercellular communication

EVs are released by most cell types and participate in local and systemic intercellular communication. In regenerative medicine, particular attention has been paid to EVs released by stem and progenitor cells, which transport proteins, lipids, and nucleic acids within a membrane-protected structure. This provides a plausible mechanism by which transplanted cells can influence injured tissues despite limited durable engraftment^[41,43]^.

MSC-derived EVs are among the most extensively studied native therapeutic EVs. Molecular and functional studies indicate that MSC-EVs can transfer regulatory RNAs to recipient cells and thereby modulate angiogenesis and other context-dependent cellular responses^[46-48]^. Proteomic studies have further identified heterogeneous protein cargoes, including extracellular-matrix-associated proteins, adhesion molecules, enzymes, and signaling-related proteins^[49]^. However, the detected proteome varies substantially with MSC source, culture conditions, EV isolation method and analytical workflow, and the functional contribution of individual proteins cannot be inferred from their detection alone^[50,51]^. Collectively, these findings support a context-dependent, multicomponent model of MSC-EV activity rather than a universal mechanism driven by a single cargo.

Experimental studies indicate that these molecular constituents can contribute to measurable biological responses. In preclinical models, MSC-EVs have ameliorated acute kidney injury^[43]^, reduced myocardial ischaemia-reperfusion injury^[41,52,53]^, promoted angiogenesis^[54,55]^ and attenuated sepsis-induced acute lung injury^[56,57]^. The molecular basis of these effects is not uniform across indications. In some models, transferred regulatory RNAs have been linked to endothelial responses and angiogenesis^[46,47,58]^, whereas in others, surface-associated enzymatic activity can make a substantial contribution, as illustrated by CD73-dependent macrophage polarization^[59]^. These findings support a context-dependent, multicomponent model of MSC-EV activity.

### The emergence of cell-free EV therapeutics

The growing recognition that EVs could mediate part of the therapeutic activity previously attributed to transplanted cells supported the development of cell-free EV therapeutics. Under this approach, purified EVs are developed as discrete, acellular biological products intended to reproduce selected functions of their parent cells without the administration of living cells.

This approach offers several translational advantages. As non-replicative entities, EVs avoid safety concerns associated with viable-cell proliferation, ectopic engraftment, and malignant transformation^[60-62]^. EVs are also more compatible than whole cells with standardized processing, dosing, formulation, cryopreservation, and off-the-shelf distribution^[63-65]^. Their properties can be adjusted through producer-cell selection and upstream bioprocess conditioning, even without deliberate bioengineering^[66-68]^.

These features explain the interest in native EVs as a link between regenerative medicine and advanced drug delivery. However, the same biological complexity that supports multifactorial activity also complicates standardization, mechanistic definition, and reproducible clinical translation.

Clinical applications of EVs include biomarker-based diagnostics and therapeutic products for regenerative, immunomodulatory, and targeted-delivery indications. Therapeutic EVs are derived from native or engineered producer cells and exert activity through either composite biological effects or introduced molecular cargoes and surface functions. Most candidates remain at the preclinical or early clinical stage, and this Review focuses on therapeutic development^[28,63-65,69]^.

## BARRIERS LIMITING NATIVE EV THERAPEUTICS

Despite these biological attributes, EV therapeutics have progressed more slowly than several other advanced modalities. As illustrated in Figure 2, therapeutic products smaller than EVs, including recombinant proteins and monoclonal antibodies, and nanoscale delivery systems of broadly comparable dimensions, such as liposomes and lipid nanoparticles (LNPs), have achieved regulatory approval or major clinical milestones^[70-73]^. Gene therapies and cell therapies have likewise established important clinical precedents^[74,75]^. EV-based therapies, by contrast, remain largely confined to preclinical and early-phase clinical development^[27,69]^.

**Figure 2:**
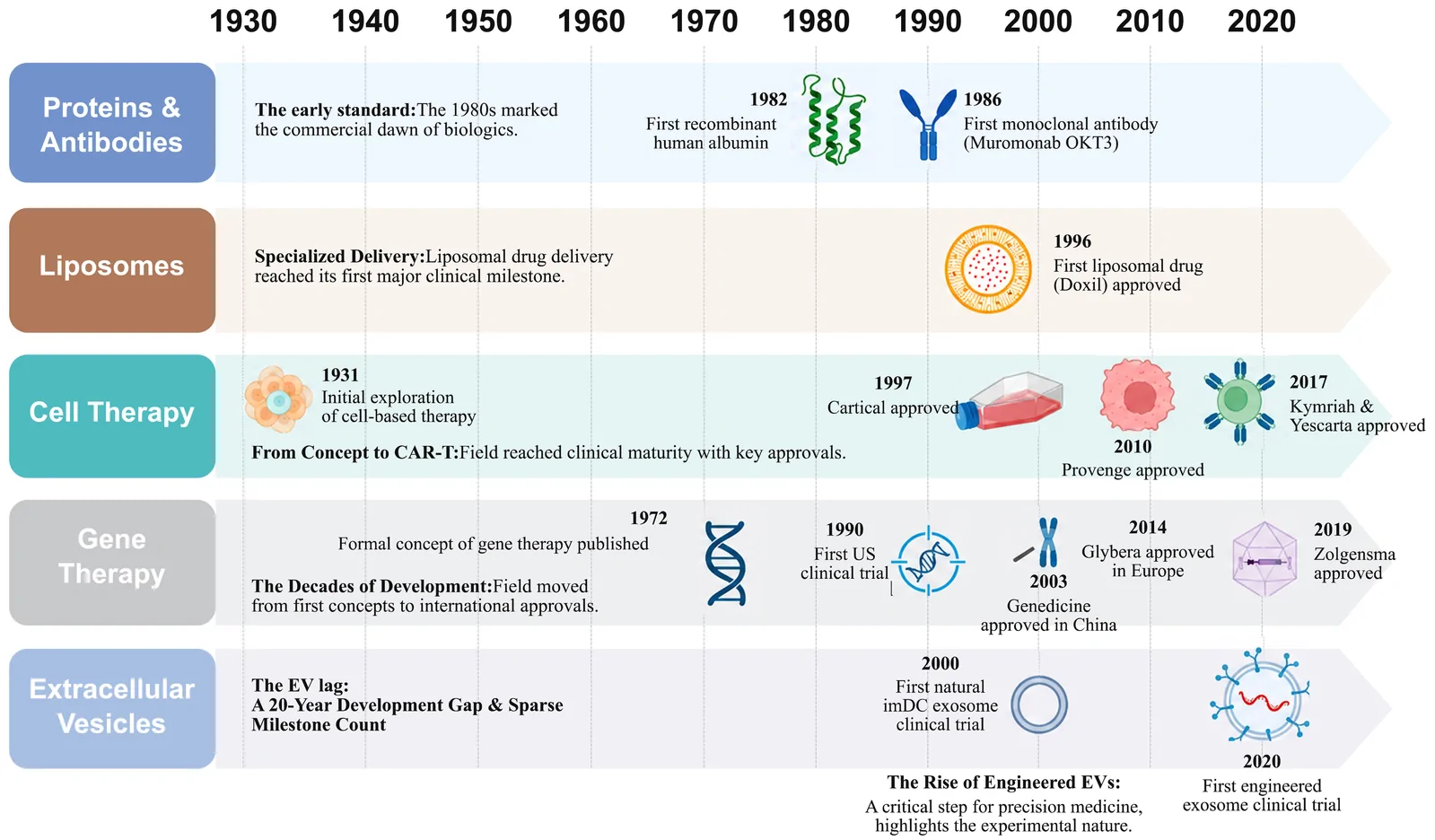
Clinical progress of therapeutic modalities highlights the translational lag of extracellular vesicles. Created with BioRender. CAR-T: Chimeric antigen receptor T-cell; EV: extracellular vesicle; imDC: immature dendritic cell.

The delayed clinical development of EV therapeutics reflects a sequence of pharmacological and product-development constraints. For an EV to deliver a cytosol-acting cargo after systemic administration, it must contain an adequate dose, remain bioavailable, reach the relevant tissue and cell population, and release functional cargo after uptake [Figure 3]. Failure at any step reduces the effective intracellular dose.

**Figure 3:**
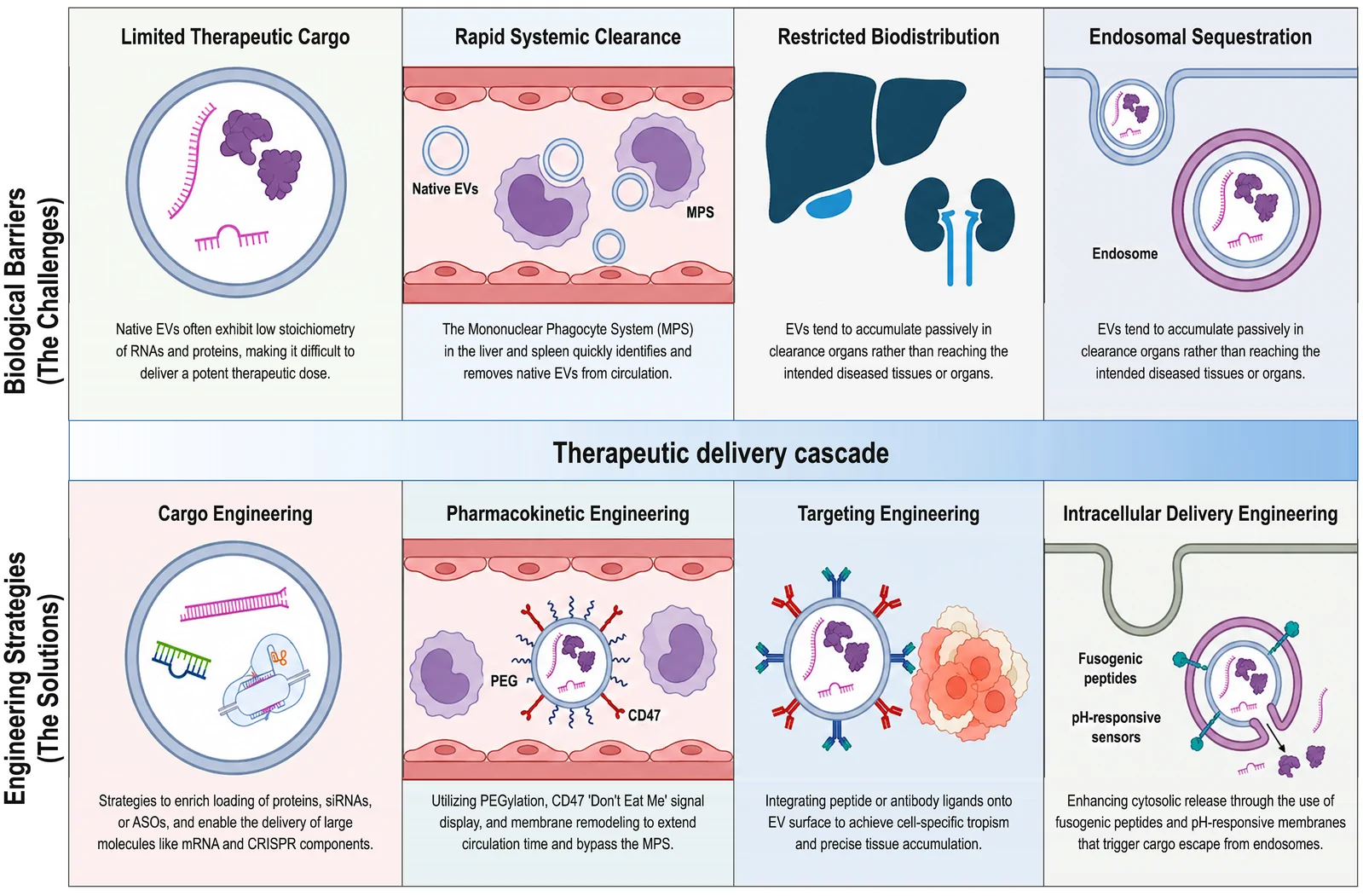
Biological barriers limiting native EV therapeutics and engineering strategies to overcome them. Created with BioRender. EVs: Extracellular vesicles; MPS: mononuclear phagocyte system; PEG: polyethylene glycol; CD47: cluster of differentiation 47; siRNAs: small interfering RNAs; ASOs: antisense oligonucleotides; mRNA: messenger RNA; CRISPR: clustered regularly interspaced short palindromic repeats; PS: phosphatidylserine.

### Stoichiometric limitations of native cargo

A fundamental limitation of native EVs is the low and heterogeneous abundance of individual therapeutic cargos. Quantitative studies have reported that many EV-associated microRNAs occur at substantially less than one copy per vesicle when averaged across bulk populations^[76,77]^. This does not exclude functional transfer by cargo-enriched EV subpopulations, but it indicates that total particle number, the proportion of cargo-positive EVs, and copies per cargo-positive vesicle are distinct attributes that should not be conflated.

Particle concentration and per-vesicle cargo stoichiometry therefore answer different questions: a preparation may contain many particles per microlitre while only a small fraction carries the molecule of interest. For therapeutic development, dose interpretation should combine particle concentration with cargo copies per EV, the fraction of cargo-positive EVs and a functional potency readout. Donor-cell state, culture conditions, and isolation procedures further alter cargo composition, complicating product standardization and definition of the active component.

### Administration routes, pharmacokinetics and systemic clearance

EV therapeutics have been administered through multiple routes, including intravenous, intratumoral, inhaled, intranasal, intrathecal, and other local or regional approaches, depending on the target tissue and intended mechanism of action^[78-80]^. The route of administration strongly influences EV exposure, biodistribution, and the biological barriers encountered after dosing. Local or compartmentalized administration may increase exposure at the intended site and partly bypass immediate systemic sequestration, whereas intravenous administration is required for many disseminated or systemically accessible indications but exposes EVs directly to blood-borne clearance mechanisms. Because quantitative pharmacokinetic data are most widely available for intravenous administration, and rapid systemic clearance is a major barrier to distal target engagement, the following discussion focuses primarily on intravenously administered EVs. Across the studies summarized in a recent systematic review, approximately 30% of injected small EV-associated signal remained in circulation at 2 min, falling to about 1.8%-3.3% at 5-30 min and approaching zero by 1 h^[78,81]^. Reported distribution-phase half-lives ranged from 1.5 to 19.9 min, indicating that the circulating fraction available for distal target engagement is often short-lived.

Rapid sequestration is therefore an upstream constraint on systemic targeting. Surface ligands cannot substantially influence distal biodistribution if most of the administered EV dose is removed before sufficient target contact occurs. Local and regional routes may reduce dependence on systemic circulation but introduce distinct barriers related to tissue retention, diffusion, mucus penetration, formulation, and compatibility with delivery devices. These route-dependent differences support a context-specific approach to EV development, in which the administration strategy is selected according to the target compartment and pharmacological constraints rather than treating intravenous delivery as a universal default.

Interpretation is also affected by the tracking method. Lipophilic fluorescent dyes, radiolabels, genetically encoded luminescent reporters, magnetic resonance tracers and cargo-associated labels may report different components of an EV preparation^[79,81,82]^. A membrane label may persist after vesicle disruption, whereas a protein or cargo reporter may be degraded after uptake. Reported pharmacokinetic curves may therefore combine intact EV disposition, fragment redistribution, and intracellular reporter metabolism^[81]^.

Most *in vivo* studies quantify blood or organ-associated signal rather than the complete metabolic fate of intact EVs. The identities of recipient cell subsets, rates of lysosomal degradation, recycling or excretion, and the relative contribution of intact vesicles and degraded material remain incompletely resolved^[79,81]^. These uncertainties limit mechanistic interpretation and complicate comparisons among formulations.

Overall, systemic EV development requires both improved blood persistence and measurement approaches that distinguish intact-vesicle exposure from redistributed labels. These considerations should be addressed before attributing changes in organ signal to active targeting.

### Tissue accumulation and the limits of native tropism

Evidence that culture-derived native EVs consistently home to specific organs remains limited. Tumour- or brain-associated accumulation varies substantially with producer-cell source, particle size, administered dose, disease model, and tracking method^[77,83-86]^. More broadly, tumour-associated accumulation of nanoscale therapeutics is also shaped by vascular permeability, interstitial transport and other tumour-microenvironment constraints^[87-89]^, providing important context for interpreting tumour-associated EV signals without establishing EV-specific active tropism. Such findings should therefore be interpreted cautiously unless supported by quantitative target-to-nontarget ratios, identification of recipient cell subsets, and evidence of functional cargo delivery.

This does not exclude organ-specific effects of disease-associated EVs. For example, CEMIP-enriched exosomes from brain-metastatic tumour cells remodelled the brain vascular niche and promoted metastatic colonization^[90]^. Such effects arise within a defined pathological context and should not be directly extrapolated to the systemic behaviour of isolated therapeutic EV preparations.

Source-dependent membrane proteins, lipids and glycans may influence cellular interactions and modify the relative distribution of EVs. However, these effects operate within the constraints imposed by short circulation, phagocytic capture and tissue accessibility. They are therefore better regarded as modulators of disposition than as evidence of strong and universally reproducible organ-selective homing.

Consistent with these constraints, unmodified EVs administered intravenously commonly accumulate in the liver, spleen, lungs and kidneys. Small EVs frequently generate prominent hepatic signals, whereas larger vesicles may show greater early pulmonary retention^[78,91]^. These patterns indicate major contributions from first-pass effects, vascular filtration and uptake by the mononuclear phagocyte system (MPS).

Bulk organ-associated signal should not be interpreted as evidence of productive targeting. Hepatic accumulation frequently reflects sequestration by Kupffer cells rather than delivery to hepatocytes^[78,84,92]^, while pulmonary signal may result from capillary retention. Detection of an EV-associated label within an organ also does not establish that intact vesicles have reached the therapeutically relevant cell population or released functional cargo. In this Review, productive targeting refers to preferential delivery to a defined tissue or cell population accompanied by measurable cargo-dependent biological activity.

Overall, the systemic distribution of intravenously administered native EVs is dominated by clearance physiology, although source-dependent features may modify relative distribution. Demonstrating therapeutically meaningful tropism therefore requires evidence beyond bulk organ accumulation, including target-cell engagement and functional delivery. These limitations provide the pharmacological rationale for the circulation-control and targeting strategies discussed in Section “Engineering extracellular vesicles for therapeutic delivery”.

### Intracellular trafficking and endosomal sequestration

EV binding and uptake can occur through clathrin-mediated endocytosis, macropinocytosis, phagocytosis, and lipid-raft-associated pathways, depending on EV size, membrane composition, surface molecules, and recipient-cell identity^[93-97]^. These entry routes influence whether EV components are recycled, degraded in lysosomes, retained within endosomes, or released to the cytosol.

Cellular uptake is therefore not equivalent to functional delivery. Cytosol-acting cargos such as siRNA, mRNA, and genome-editing complexes must escape endosomal confinement, and this requirement becomes more stringent when only a small fraction of the administered dose reaches the target tissue or carries the intended cargo.

EVs and ionizable LNPs differ in both composition and intracellular delivery logic. EVs are generated by cells and contain a heterogeneous protein-lipid membrane, whereas LNPs are assembled from defined synthetic lipids during formulation. Ionizable LNPs rely mainly on pH-dependent lipid protonation and endosomal membrane destabilization; quantitative imaging has estimated that only a small fraction of internalized siRNA reaches the cytosol^[98,99]^. EV cargo release has instead been proposed to involve direct membrane fusion or back-fusion with the endosomal limiting membrane^[95,96,99,100]^. Available studies suggest that EVs can release luminal cargo through non-lytic endosomal mechanisms without extensive disruption of endosomal integrity and may therefore offer advantages over LNPs in specific experimental settings. More quantitative head-to-head comparisons and mechanistic studies are needed to determine the magnitude and generalizability of this potential advantage.

Back-fusion provides a plausible non-lytic route for cytosolic release, but current evidence remains methodologically heterogeneous^[95,96,99,100]^. Mechanistic studies should distinguish surface binding, uptake, endosomal retention, membrane fusion, and functional cargo release rather than inferring all steps from bulk intracellular fluorescence.

This distinction is important for engineering studies: increased cell-associated signal may reflect stronger binding or prolonged endosomal retention without increasing cytosolic exposure. Functional reporter assays, split-protein systems, subcellular imaging, and pharmacodynamic readouts are therefore required to establish productive delivery.

For cytosol-acting EV therapeutics, the relevant endpoint is functional cargo access to its intracellular target. Endosomal escape should be treated as a product-specific critical attribute rather than assumed from uptake alone.

## ENGINEERING EVs FOR THERAPEUTIC DELIVERY

The pharmacological limitations described above - including low and heterogeneous cargo abundance, rapid systemic clearance, clearance-dominated tissue distribution, and inefficient intracellular cargo release - provide the rationale for EV engineering. As summarized in Figure 3, engineering strategies have been developed to address these barriers by increasing cargo loading, prolonging systemic exposure, improving tissue- or cell-selective engagement, and enhancing functional intracellular delivery.

These interventions can be introduced at two broad stages: before EV isolation through producer-cell modification and cargo recruitment during EV biogenesis, or after isolation through cargo loading, surface functionalization, or hybrid-particle formation^[27,33,34,101]^ [Figure 4 and Table 1]. The choice of strategy should be guided by the specific pharmacological barrier to be addressed, while also considering its effects on product heterogeneity, immunogenicity, process recovery, and scalability.

**Figure 4:**
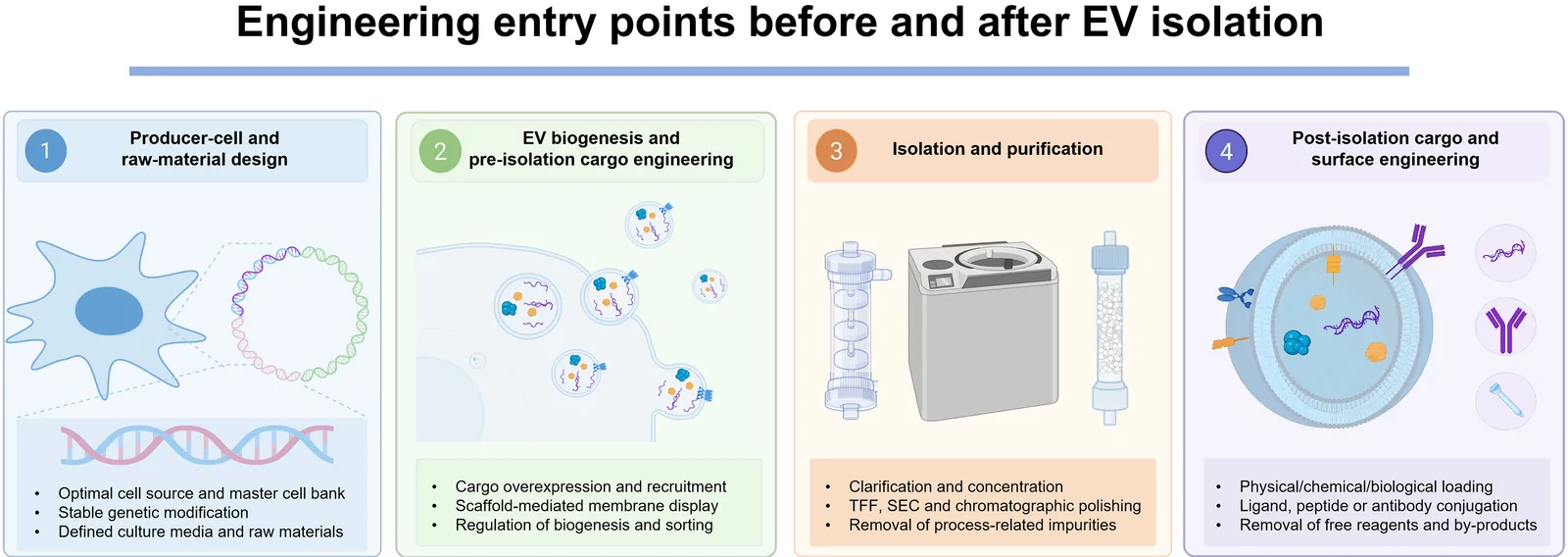
Engineering entry points before and after EV isolation. Producer-cell and raw-material design establishes the manufacturing system; pre-isolation engineering modifies cargo recruitment and EV biogenesis; isolation and purification remove process-related impurities; and post-isolation engineering loads cargo or functionalizes the EV surface. Created with BioRender. EV: Extracellular vesicle; TFF: tangential flow filtration; SEC: size-exclusion chromatography; CMC: chemistry, manufacturing and controls.

**Table 1:** Major engineering strategies for therapeutic EV development

**Engineering layer**	**Representative approaches**	**Principal strengths**	**Main limitations or risks**	**Representative references**
Pre-isolation cargo engineering	Cargo overexpression; scaffold-mediated protein loading; RNA-binding motifs	Integrated loading during biogenesis; compatible with stable cell banks	Variable loading stoichiometry; effects on producer-cell biology; clone-to-clone variability	[30,102,103]
Post-isolation cargo loading	Electroporation; sonication; extrusion; transient permeabilization	Applicable to synthetic cargos and purified EVs; modular	Aggregation, membrane damage, cargo precipitation and surface adsorption artefacts	[34,99,101,104]
Circulation and anti-phagocytic engineering	PS reduction or masking; CD47 display; PEGylation	Can increase the bioavailable fraction and reduce early mononuclear phagocyte system uptake	Stealth-uptake trade-off; altered biodistribution; anti-PEG or other immune responses	[86,105-108]
Targeting moieties	Peptides; nanobodies; scFvs; aptamers; antibodies	Peptides permit high display density; nanobodies combine compact size and affinity; scFvs offer broad antibody specificity	Proteolytic instability, folding or aggregation, orientation control, immunogenicity and manufacturing complexity	[23,109-117]
Intracellular-delivery engineering	VSV-G; syncytins; fusogenic peptides; membrane-lipid remodelling	Can enhance membrane fusion and functional cytosolic release	Potential immunogenicity, altered membrane integrity and cell-type-dependent activity	[100,118-120]
Hybrid biological-synthetic systems	EV-LNP or EV-liposome fusion; EV-enveloped viral vectors	Combines biological surface functions with synthetic loading and formulation control	Hybrid identity, fusion efficiency, residual unfused particles and complex CMC characterization	[121-123]

The selection of an engineering method requires a balance among functional gain, product heterogeneity, immunogenicity, scalability and analytical control. PS: Phosphatidylserine; CD47: cluster of differentiation 47; PEG: polyethylene glycol; scFvs: single-chain variable fragments; VSV-G: vesicular stomatitis virus glycoprotein; EV: extracellular vesicle; LNP: lipid nanoparticle; CMC: chemistry, manufacturing and controls.

### Cargo loading and hybrid delivery systems

Cargo engineering aims to increase the abundance and reproducibility of active molecules per EV. Pre-isolation strategies use scaffold proteins, membrane-associated sorting domains, or RNA-binding modules to enrich proteins and nucleic acids during biogenesis^[30,102,103]^. These approaches can support stable manufacturing cell lines, but loading efficiency, topology, and effects on producer-cell physiology require direct measurement.

Post-isolation approaches, including electroporation, sonication, extrusion and transient permeabilization, broaden the range of deliverable synthetic cargos but may promote aggregation, membrane disruption or apparent loading caused by surface adsorption^[101,104]^. Endogenous and exogenous loading should therefore be compared using recovery, integrity, cargo localization, and functional delivery metrics rather than nominal loading alone.

Engineered EVs are now being evaluated for mRNA, proteins, CRISPR-Cas ribonucleoproteins (RNPs) and virus-associated cargos^[121,122,124-127]^. Hybrid EV-LNP systems provide a complementary approach: low-pH fusion of EVs with mRNA-loaded LNPs generated hybrid vesicles with functional mRNA delivery and predominant splenic activity in mice^[123]^. Such hybrids may combine EV surface biology with synthetic loading efficiency, but they introduce additional requirements for defining fusion efficiency, residual unfused particles, and hybrid-particle identity.

Together, these studies extend EV engineering beyond small RNAs to large mRNAs, CRISPR-Cas RNPs, base editors and adeno-associated virus (AAV)-vesicle hybrids, while also increasing product-definition and manufacturing complexity^[121,122,126,127]^.

### Engineering systemic circulation and stealth properties

Native EVs are rapidly removed from blood, largely through macrophage uptake in the liver and spleen^[78,84,85]^. Circulation engineering therefore aims to reduce early non-productive recognition while retaining uptake by the intended target cells.

Phosphatidylserine (PS) exposure and negative surface charge contribute to macrophage recognition, whereas PS-deficient EV subpopulations show prolonged retention^[106,107]^. CD47-SIRPα signalling and steric shielding with polyethylene glycol (PEG) have also been used to reduce phagocytic uptake^[86,105]^. An engineered small extracellular vesicle (sEV) product combining an epidermal growth factor receptor (EGFR)-targeting nanobody, CD47, and miR-204-5p extended circulation and produced antitumour activity in EGFR-positive models, illustrating how anti-phagocytic and targeting functions can be integrated within one product^[108]^.

These modifications require a balance between stealth and productive uptake. Excessive masking may reduce target-cell internalization, while altered surface composition may change complement activation, tissue distribution or repeat-dose immunogenicity. Circulation time should therefore be interpreted together with intact-EV exposure, target-cell delivery and pharmacodynamic activity.

Prolonged circulation does not guarantee efficacy, but it can increase the EV fraction available for subsequent targeting and intracellular delivery. It should be treated as an enabling pharmacokinetic attribute rather than an independent therapeutic endpoint.

### Engineering tissue- and cell-selective targeting

Active targeting is meaningful only for the EV fraction that remains bioavailable. Targeting should therefore be evaluated together with circulation, organ-level exposure and target-cell engagement rather than as an isolated surface feature.

Targeting functions may be introduced by producer-cell expression of peptides, nanobodies, single-chain variable fragments (scFvs) or other binders on EV-associated membrane scaffolds, or by post-isolation lipid insertion and covalent or non-covalent conjugation^[23,86,109-111,113-116]^. Peptides are compact and permit high surface density but may have limited affinity or proteolytic stability. Nanobodies are small and stable but may require humanization; scFvs provide broad antibody-derived specificity but can present folding, aggregation, and orientation challenges. The optimal ligand therefore depends on target density, required affinity, surface topology, immunogenicity, and manufacturability [Table 1].

Early proof-of-concept studies showed that rabies virus glycoprotein (RVG) peptide-displaying EVs could enhance siRNA delivery to neural tissues following systemic administration, demonstrating that engineered surface ligands can modify EV distribution and therapeutic exposure^[23]^. More recent studies have shifted the emphasis from organ-level signal toward receptor-dependent delivery to defined cellular compartments. NBsEV204 combined an EGFR-targeting nanobody with CD47 and miR-204-5p and linked tumour-cell recognition and prolonged exposure to antitumour activity in EGFR-positive models^[108]^. Dual nanobody-engineered milk EVs were designed to engage both tumour-associated macrophages (TAMs) and malignant cells, coupling multi-cell targeting to microenvironmental reprogramming and therapeutic activity^[111]^. CD3ε nanobody-engineered EVs delivered a CAR.BiTE transgene to CD3-positive T cells *in vivo* and generated functional T-cell responses rather than merely increasing signal in a target organ^[117]^. Antigen-displaying EVs that enhance chimeric antigen receptor T-cell (CAR-T-cell) activity provide a related example in which both the recipient-cell compartment and the downstream function are explicitly defined^[109,110]^.

Together, these studies support a hierarchy of targeting evidence: receptor-dependent binding or uptake, enrichment in the intended cell subset, cargo-specific pharmacodynamic change, and disease-relevant functional benefit. The strongest targeting claims therefore move beyond bulk organ accumulation toward functional delivery to defined cellular compartments.

Targeting claims should be supported by quantitative target-to-nontarget ratios, receptor-dependent controls, identification of recipient cell populations and downstream pharmacodynamic activity. Bulk fluorescence alone cannot distinguish active binding from passive trapping or phagocytic uptake.

### Engineering intracellular delivery and endosomal escape

For EVs carrying cytosol-acting RNAs, proteins or genome-editing complexes, uptake must be followed by functional cargo release. EVs may use membrane-fusion-dependent pathways that differ from the pH-responsive membrane destabilization of ionizable LNPs, but the efficiency of EV-mediated escape remains strongly dependent on cell type, cargo, and assay design^[95,96,99]^.

Viral fusogens such as vesicular stomatitis virus glycoprotein (VSV-G) can markedly increase cytosolic cargo delivery, and endogenous or retroviral-derived proteins such as syncytins provide non-VSV-G alternatives^[100,118,119]^. Membrane-lipid composition may also influence fusion; EV-associated cholesterol contributes to membrane organization and biological activity, although direct evidence that deliberate lipid remodelling consistently improves endosomal escape remains limited^[120]^.

However, VSV-G is not immunologically inert. VSV-G-displaying retrovirus-like particles induce neutralizing IgM and class-switched IgG responses^[128]^, and incorporation of fusion-competent VSV-G into exosome-like vesicles enhances dendritic-cell uptake, maturation, and antigen cross-presentation^[129]^. In VSV-G-pseudotyped lentiviral systems, pre-existing anti-envelope immunity markedly suppresses homologous redosing, whereas serologically distinct vesiculovirus G proteins can partly restore transduction^[130]^. Thus, virus-like particle (VLP) and pseudotyped-vector studies clearly establish that VSV-G can induce neutralizing anti-envelope immunity. Direct repeated-dose evidence for therapeutic VSV-G-engineered EVs remains limited, but the risk is supported by strong biological and cross-platform evidence.

The objective is therefore not simply to maximize endosomal disruption, but to increase functional delivery while preserving EV integrity and acceptable safety. Split-reporter assays, cytosolic cargo sensors, and pharmacodynamic readouts are preferable to uptake measurements alone.

Engineering can also introduce new immunogenicity risks. Repeat-dose programmes using VSV-G should evaluate VSV-G-specific binding and neutralizing antibodies, complement activation, altered clearance, and loss of functional exposure^[128-130]^. Similar considerations apply to non-human scFvs or nanobodies, for which protein origin, humanization, surface density, pre-existing immunity and dosing schedule should guide candidate selection. Complement assays, human immune-cell testing, cytokine-release assessment and repeat-dose immunogenicity studies should therefore be incorporated into development plans^[33,34]^.

## TRANSLATIONAL PROGRESS AND LESSONS FROM ENGINEERED EV THERAPEUTICS

Table 2 summarizes representative engineered EV programmes across preclinical and clinical stages and is intended to highlight translational patterns rather than provide an exhaustive pipeline inventory. Three features are apparent. First, clinically tested products are concentrated in immune-facing, locally delivered or compartment-specific applications. Second, differentiated preclinical systems increasingly combine targeting, cargo loading, and circulation control rather than relying on a single modification. Third, clinical entry has not removed constraints related to dose, manufacturing complexity, repeat administration or commercial sustainability. The following sections therefore organize recent progress by functional objective before considering programme-level clinical lessons.

**Table 2:** Representative engineered EV programmes spanning clinical-stage products and preclinical platform concepts

**Programme**	**EV source**	**Engineering/active component**	**Route**	**Development status**	**Indication/function**	**Translational note and reference**
exoSTING	Engineered producer-cell EVs	Loaded STING agonist	Intratumoral	Phase I; planned Phase II paused	Solid tumours; antigen-presenting-cell activation	Local delivery generated early clinical experience; programme paused during Codiak reprioritization^[31,135]^
exoIL-12	Engineered producer-cell EVs	Surface-displayed IL-12	Intratumoral	Phase I; planned Phase II paused	Solid tumours; local T-cell activation	Illustrates spatial restriction of a potent cytokine; programme paused^[30,135]^
exoASO-STAT6	Engineered producer-cell EVs	STAT6 antisense oligonucleotide	Intravenous	Phase I initiated; subsequent continuity uncertain after restructuring	Tumour-associated macrophage reprogramming in liver tumours	Systemic myeloid-cell-directed programme^[32,135,139]^
iExoKrasG12D	MSC-derived EVs	KRASG12D siRNA	Intravenous	Phase I; early results reported	Metastatic pancreatic cancer	Demonstrated clinical testing of systemic EV-mediated RNA delivery^[136]^
ILB-202	Engineered HEK293 EVs	EXPLOR-loaded super-repressor IκBα	Intravenous	Phase I completed; single ascending dose	NF-κB inhibition; inflammatory diseases	Eighteen healthy volunteers; no serious or dose-limiting toxicity; exploratory pharmacodynamic signals^[137]^
EXO-CD24	Engineered-cell exosomes	Surface-enriched CD24	Inhalation	Randomized dose-finding Phase IIb reported	COVID-19-associated respiratory disease	Ninety-one patients; no treatment-related adverse events; efficacy interpretation limited by the absence of a placebo arm^[138]^
CD3-targeted CAR.BiTE EVs	Engineered producer-cell EVs	CD3 nanobody plus CAR.BiTE transgene	Intravenous	Preclinical	*In vivo* T-cell reprogramming	Functional targeting of a defined immune-cell compartment^[117]^
NBsEV204	Engineered-cell sEVs	EGFR nanobody, CD47 and miR-204-5p	Intravenous	Preclinical	EGFR-positive tumours	Integrates targeting, anti-phagocytic engineering and cargo loading^[108]^
Dual-nanobody milk EVs	Milk-derived EVs	Two nanobody targeting functions	Intravenous	Preclinical	Tumour cells and tumour-associated macrophages	Example of simultaneous multi-cell targeting^[111]^
Hybrid EV-LNP mRNA system	Cell-derived EVs fused with mRNA-LNPs	mRNA-loaded hybrid EVs	Intravenous	Preclinical	Functional mRNA delivery	Combines EV surface features with LNP loading and escape properties^[123]^
exo-AAV/EV-enveloped AAV	Producer-cell EV-viral hybrids	AAV capsids associated with or enclosed by EVs	Intravenous/local	Preclinical/early translational	Gene delivery	Potential antibody shielding but increased product-definition complexity^[121,122]^
RVG-engineered EVs	Dendritic-cell-derived EVs	RVG peptide plus siRNA	Intravenous	Preclinical; historical proof of concept	Central nervous system delivery	Landmark demonstration of ligand-directed EV delivery^[23]^

Development statuses reflect publicly reported information available to the authors during manuscript revision and may change with subsequent clinical or regulatory updates. EV: Extracellular vesicle; STING: stimulator of interferon genes; IL-12: interleukin-12; ASO: antisense oligonucleotide; STAT6: signal transducer and activator of transcription 6; MSC: mesenchymal stem cell; KRAS: KRAS proto-oncogene, GTPase; siRNA: small interfering RNA; HEK293: human embryonic kidney 293; EXPLOR: exosomes for protein loading via optically reversible protein-protein interactions; IκBα: inhibitor of nuclear factor kappa B alpha; NF-κB: nuclear factor kappa B; CD24: cluster of differentiation 24; CD3: cluster of differentiation 3; CAR: chimeric antigen receptor; BiTE: bispecific T-cell engager; sEV: small extracellular vesicle; EGFR: epidermal growth factor receptor; CD47: cluster of differentiation 47; miR: microRNA; LNP: lipid nanoparticle; mRNA: messenger RNA; AAV: adeno-associated virus; RVG: rabies virus glycoprotein; CNS: central nervous system.

This selective development model does not imply that one EV platform will replace all synthetic carriers. Instead, it emphasizes settings in which biological membrane functions, route-specific delivery, or cell-selective engagement provide a measurable advantage over an appropriate comparator.

### From proof-of-concept targeting to context-specific development

Early engineered EV studies emphasized systemic targeting, including delivery to the central nervous system^[23]^. Subsequent work has shown that translational performance depends on the combined effects of administration route, pharmacokinetics, cargo stoichiometry, intracellular release, and scalable manufacture^[69,76,78,91,99,131,132]^.

Accordingly, local or regional administration, compartment-restricted disease and immune-facing applications have gained prominence. These settings can reduce dependence on long systemic circulation and align the EV design with a defined biological barrier or recipient-cell population.

### Macromolecular and multimodal delivery

Engineered EVs have expanded beyond small-RNA transport to the delivery of large proteins and genome-editing machinery. Platforms that combine cargo loading with fusogenic engineering have delivered Cre recombinase, Cas9/sgRNA ribonucleoproteins and anti-inflammatory proteins, including functional reporter recombination after intracerebroventricular administration^[127]^. Hybrid EV systems and multimodal EVs further illustrate the potential to integrate targeting, surface signalling and internal cargo within one construct^[123,133,134]^.

The translational value of these systems lies in enabling a defined function that is difficult to reproduce with native EVs, rather than in incremental increases in organ-associated signal. However, each added function increases product complexity and requires quantitative control of cargo abundance, surface topology, and functional delivery.

### Precision immune-cell reprogramming: engineering routes and functional endpoints

Precision immune-cell reprogramming represents a context-specific opportunity for engineered EVs because both the recipient-cell population and the desired functional state can be defined. Current approaches include receptor-directed cargo delivery, surface-mediated immune activation, and combined targeting-cargo systems.

CD3-targeted EVs have been used to deliver CAR.BiTE constructs to T cells *in vivo*^[117]^, whereas antigen-displaying EVs can enhance CAR-T cell activation or reduce the consequences of antigen escape^[109,110]^. Myeloid-directed approaches include exoASO-STAT6, which was designed to deliver an antisense oligonucleotide to TAMs and suppress immunosuppressive STAT6 signalling^[32]^. Related strategies include STING-agonist delivery to antigen-presenting cells (APCs) and surface-displayed interleukin-12 (IL-12) for spatially restricted T-cell activation^[30,31]^.

Although these systems use different engineering mechanisms, they share a common translational requirement: evidence that the EV reaches the intended immune-cell subset and produces a defined change in cellular state or function. Relevant endpoints include cell-selective exposure, pathway modulation, durable phenotypic change, and acceptable systemic immune activation. Several immune-directed designs have progressed into clinical testing and are considered below from a programme-development perspective.

### Clinical translation, setbacks and programme-level lessons

Several immune-directed designs described above have entered clinical testing, making them informative case studies of pharmacological feasibility, dose selection, manufacturing execution and programme sustainability. Codiak BioSciences advanced multiple engineered exosome products into Phase I studies, including intratumoral exoSTING and exoIL-12 and systemically administered exoASO-STAT6^[30-32,135]^. These programmes generated early safety and pharmacodynamic observations, but planned Phase II studies of exoSTING and exoIL-12 were paused during a 2022 corporate reprioritization^[135]^.

The Phase I iExoKrasG12D study provided evidence that systemically administered, siRNA-loaded MSC-derived EVs could be evaluated in metastatic pancreatic ductal adenocarcinoma, with an acceptable early safety profile and pharmacodynamic signals reported in a subset of patients^[136]^. A further milestone is ILB-202, a Korean-developed HEK293-EV product loaded with a super-repressor form of IκBα through the light-inducible EXPLOR system. In a randomized, double-blind, placebo-controlled, single-ascending-dose Phase I trial, 18 healthy volunteers received intravenous ILB-202. No serious or dose-limiting toxicities were reported, although mild reductions in natural killer-cell counts and one grade 1 neutropenia event were observed; single-cell transcriptomics provided exploratory evidence of nuclear factor kappa B (NF-κB)-associated pharmacodynamic modulation^[137]^.

EXO-CD24 represents a distinct route-aligned design. The product uses exosomes engineered to overexpress surface CD24 and was administered by inhalation for five consecutive days in a randomized, single-blind, dose-finding Phase IIb study involving 91 patients with mild-to-moderate COVID-19-related acute respiratory distress syndrome. No treatment-related adverse events were reported, and respiratory and inflammatory measures improved; however, the study compared two active dose levels without a concurrent placebo arm, so efficacy requires confirmation in a larger controlled trial^[138]^.

Clinical entry does not by itself establish commercial sustainability. Codiak subsequently filed for voluntary Chapter 11 protection and pursued an asset sale in 2023^[139]^. The sequence from early clinical testing to programme pauses and corporate restructuring highlights the combined importance of efficacy magnitude, dose, manufacturing cost, capital requirements and portfolio prioritization. These setbacks should be interpreted as programme-level lessons rather than evidence that the EV modality is either validated or invalidated as a whole.

Across the programmes summarized in Table 2, the most credible advances are those in which the administration route, recipient-cell population, engineering function and pharmacodynamic endpoint are jointly defined. Local delivery, immune-cell reprogramming and complex cargo transport currently provide the clearest examples of such alignment. Engineered EV products should therefore be compared with relevant alternatives using route-specific exposure, functional delivery, safety, manufacturability and cost.

## FUTURE PERSPECTIVES

Future development should convert the programme-level lessons above into product-specific design, regulatory and manufacturing principles. The central goal is reproducible performance in defined tissues, routes or cellular compartments rather than platform-wide superiority.

### From context-specific success to reusable EV platforms

The translational lessons summarized in Section 5 suggest that EV platform development should begin with a biologically aligned and clinically defined use case rather than a claim of universal delivery. The administration route, target compartment, recipient-cell population, and intended mechanism of action should be specified together, and performance should be evaluated against an appropriate therapeutic or delivery comparator.

Platform reuse should be considered only after a delivery design reproducibly achieves intact-vesicle exposure, target-cell engagement and functional cargo activity. At that stage, different cargos may be evaluated using a shared producer-cell, manufacturing and delivery framework. Figure 5 illustrates this progression from broad carrier ambitions toward validated, context-specific platforms that may subsequently support multiple therapeutic cargos.

**Figure 5:**
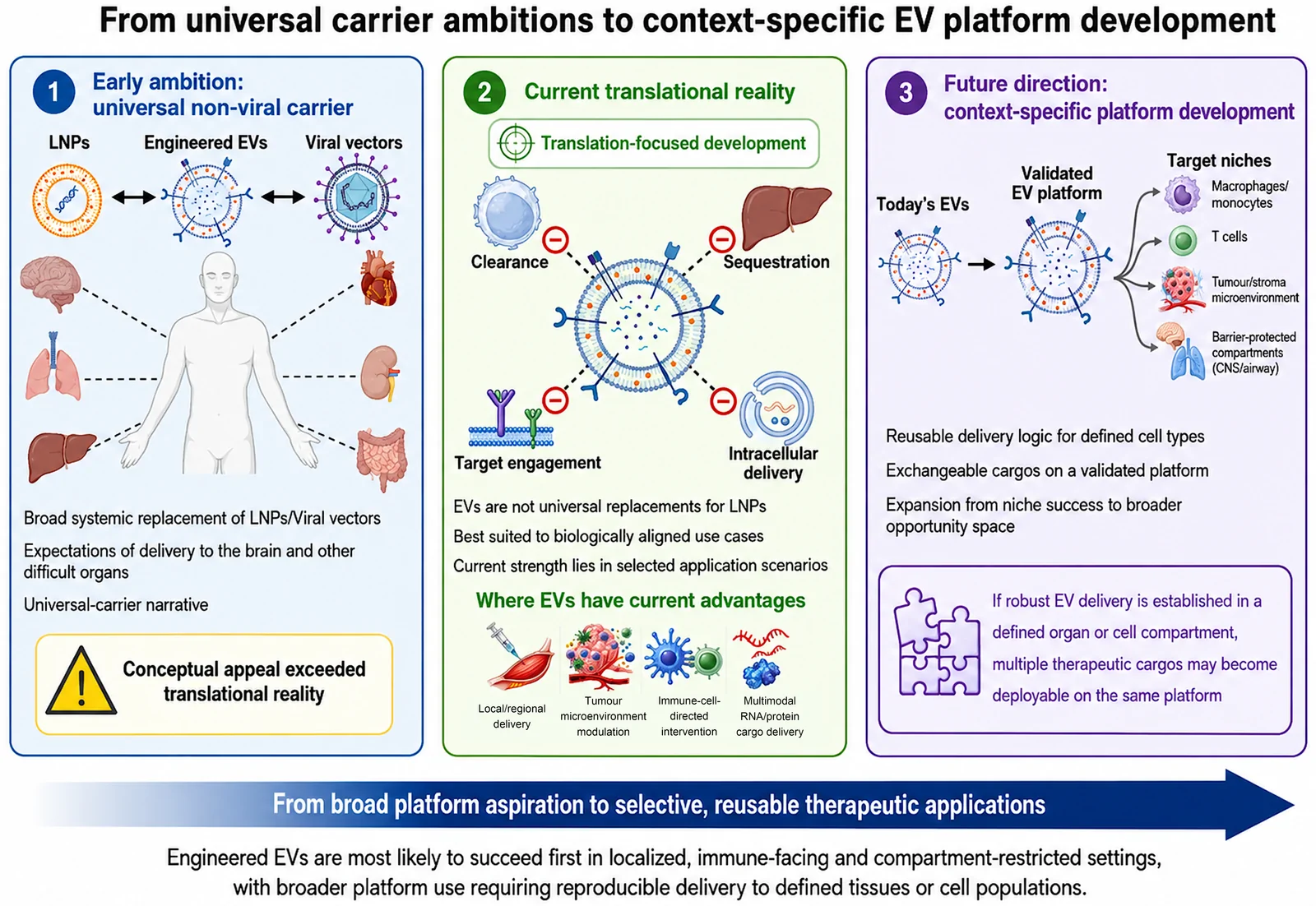
From universal carrier ambitions to context-specific EV platform development. Development should begin with a defined route, tissue, or cell compartment; broader platform reuse becomes credible only after delivery and functional activity are reproducibly established. Created with BioRender. EV: Extracellular vesicle; LNPs: lipid nanoparticles; CNS: central nervous system.

### Mechanism-based product and potency design

Once a clinically defined use case has been selected, engineering parameters should be optimized according to the intended mechanism of action rather than by maximizing EV uptake or a single product attribute. EVs can signal through transient surface engagement, prolonged membrane association, or uptake-dependent cargo transfer, and the relevant mode depends on the intended mechanism of action.

As observed for antibodies and CAR-T cells, maximizing a single engineering parameter can reduce overall performance. Very high affinity may impair tissue penetration or target-density discrimination, while excessive internalization may be undesirable for a surface-signalling product. EV design should therefore begin with a causal model linking surface topology, cargo localization, cellular interaction and pharmacodynamic activity.

For cytosolic cargos, loading, uptake and endosomal release are primary constraints. For surface-acting products, ligand density, orientation, residence time and receptor clustering may be more important. This distinction should guide potency-assay design and comparability studies.

### Function-based regulatory evaluation

Research-reporting frameworks such as MISEV2014, MISEV2018 and MISEV2023 provide a common foundation for EV characterization^[82,140,141]^, but they do not by themselves define a pharmaceutical control strategy. Engineered EVs can resemble recombinant biologics, cell-derived medicinal products, synthetic nanocarriers or hybrid constructs. Classification based only on cellular origin is therefore unlikely to capture the risks and active components of every product. Regulatory evaluation should therefore be driven by the introduced function and its associated product risks, rather than by the presence of an EV membrane alone.

A function-based assessment should identify the active component, intended mechanism of action and engineering-associated risks. Cytokine-displaying EVs, genome-editor-delivering EVs and receptor-clustering EVs require different potency assays, identity attributes and comparability strategies even though they share a vesicular membrane.

Regulatory convergence will depend on clearer causal links between product attributes and biological activity. Mechanistic uncertainty directly limits the ability to define release criteria, acceptable variability and clinically relevant comparability.

### Manufacturing, cost, and supply-chain readiness

Manufacturing readiness should be evaluated against the intended dose, dosing frequency and number of clinically relevant doses produced per batch, rather than culture volume alone. Raw-material control begins with qualified cell banks and defined media. Native MSC-EVs require control of tissue and donor source, passage history, xeno-free supplements, microcarriers and collection conditions; engineered EVs add expression constructs, selection, transfection or coupling reagents, together with evidence that the engineered phenotype remains stable^[131,132,142-144]^.

Upstream output reflects viable producer-cell mass, EV productivity per cell, harvest frequency and potency per particle. Planar systems may support early or low-dose local-delivery products, whereas repeated systemic dosing is more likely to require high-density bioreactor or perfusion processes. Scale should therefore be reported as conditioned-medium volume, total recovered particles or potency units, overall process recovery and clinically relevant doses generated per lot^[142,143,145]^. These variables, rather than nominal reactor size, determine cost of goods and the feasibility of qualified, preferably redundant, raw-material and single-use supply chains.

Downstream processing must balance purity, recovery and throughput. Tangential flow filtration (TFF) is useful for concentration and diafiltration and is often combined with chromatographic polishing, but every additional unit operation adds processing time and product loss. This is particularly important for post-isolation engineering: lipid insertion, covalent conjugation, exogenous cargo loading or EV-LNP hybridization can leave free cargo, reagents, aggregates or unmodified particles and may require a second purification or concentration step. The resulting loss in recoverable functional EVs can materially increase the required upstream batch size and cost, while adding controls for modification efficiency, residual reagents, membrane integrity and potency^[104]^. Pre-isolation engineering avoids this secondary purification but shifts risk toward cell-line stability and batch consistency.

Formulation and distribution should be developed with consideration of the dose concentration, route and container-closure system. Frozen liquid products impose cold-chain and freeze-thaw constraints. Lyophilization may improve distribution and point-of-care use, but requires product-specific optimization of protectants, cycle parameters, residual moisture, reconstitution and post-reconstitution stability; engineered EVs may not behave like native EVs when displayed proteins, inserted lipids or hybrid components alter membrane stability^[146,147]^.

### Concluding perspective

Engineered EVs are most likely to succeed when the administration route, recipient cell population, engineering function and pharmacodynamic endpoint can be jointly defined and reproducibly measured.

Broader platform value should be claimed only after a validated delivery design supports multiple cargos without compromising potency, safety or manufacturing consistency.

## DECLARATIONS

### AI and AI-assisted tools statement

During the preparation of this manuscript, the AI tools ChatGPT (OpenAI, accessed 2026-08-08) and Kimi (Moonshot AI, accessed 2026-08-08) were used solely for language editing without unsupervised automatic generation. In addition, the AI tool ChatGPT (OpenAI, accessed 2026-08-08) was used solely to assist with preparing the Graphical Abstract. The tool did not influence the study design, data collection, analysis, interpretation, or the scientific content of the work. All authors take full responsibility for the accuracy, integrity, and final content of the manuscript.

### Copyright

© The Author(s) 2026.
